# Drug Loading in Chitosan-Based Nanoparticles

**DOI:** 10.3390/pharmaceutics16081043

**Published:** 2024-08-06

**Authors:** Yedi Herdiana, Ellin Febrina, Siti Nurhasanah, Dolih Gozali, Khaled M. Elamin, Nasrul Wathoni

**Affiliations:** 1 Department of Pharmaceutics and Pharmaceutical Technology, Faculty of Pharmacy, Universitas Padjadjaran, Sumedang 45363, Indonesia; 2 Department of Pharmacology and Clinical Pharmacy, Faculty of Pharmacy, Universitas Padjadjaran, Sumedang 45363, Indonesia; ellin.febrina@unpad.ac.id; 3 Faculty of Agricultural Industrial Technology, Universitas Padjadjaran, Sumedang 45363, Indonesia; siti.nurhasanah@unpad.ac.id; 4 Graduate School of Pharmaceutical Sciences, Kumamoto University, Kumamoto 862-0973, Japan; khaled@kumamoto-u.ac.jp

**Keywords:** functionality, nanomedicines, encapsulation efficiencies, drug release, stability

## Abstract

Chitosan nanoparticles (CSNPs) are promising vehicles for targeted and controlled drug release. Recognized for their biodegradability, biocompatibility, low toxicity, and ease of production, CSNPs represent an effective approach to drug delivery. Encapsulating drugs within nanoparticles (NPs) provides numerous benefits compared to free drugs, such as increased bioavailability, minimized toxic side effects, improved delivery, and the incorporation of additional features like controlled release, imaging agents, targeted delivery, and combination therapies with multiple drugs. Keys parameters in nanomedicines are drug loading content and drug loading efficiency. Most current NP systems struggle with low drug loading, presenting a significant challenge to the field. This review summarizes recent research on developing CSNPs with high drug loading capacity, focusing on various synthesis strategies. It examines CSNP systems using different materials and drugs, providing details on their synthesis methods, drug loadings, encapsulation efficiencies, release profiles, stability, and applications in drug delivery. Additionally, the review discusses factors affecting drug loading, providing valuable guidelines for future CSNPs’ development.

## 1. Introduction

The field of nanotechnology has empowered the creation of new therapeutic strategies, marked by the emergence of targeted nano drug delivery systems, controlled release mechanisms, and the synthesis of theranostic agents [1,2]. In the context of drug delivery using NPs, a very important task is the quantification of the drug that is inserted accurately, ensuring reproducibility and reliability in therapeutic results [3,4]. NPs offer innovative solutions to the challenges inherent in traditional therapeutic modalities [5]. As we navigate the intricacies of biological barriers, from the systemic level to the microenvironment and cellular, the need for precision therapy becomes increasingly clear [6,7,8]. Precision interventions tailored to each patient’s profile have significantly increased the effectiveness of therapy [9,10,11]. The inherent advantages of this technology, including sustained drug release, targeted delivery, and impressive surface-to-volume ratio, make it a promising candidate for a transformative drug delivery system [12]. Among them, CSNPs, which take advantage of biodegradable and biocompatible polymers, offer stability, non-toxicity, and ease of preparation [13]. NPs in drug delivery systems have experienced remarkable progress, enhancing therapeutic results and patient experiences [14].

Advances in nanomedicine have not only revolutionized drug formulation but also brought advances in bioavailability, reduced toxicity, and controlled release. In particular, the integration of hydrophobic drugs into nanomedicines has shown remarkable improvements in solubility, stability, efficacy, and minimized side effects. The multifunctionality of nanomedicine, which includes imaging, triggered release, and targeted delivery, is increasingly highlighting its versatility [15]. The choice between synthetic and natural polymers introduces an important dimension to the synthesis method, with natural polymers standing out due to their biocompatibility, biodegradability, and ease of surface modification [16,17,18].

CSNPs are increasingly used due to their efficient payload capacity, controlled drug release, and enhanced bioavailability [19]. Polymeric nanocarriers excel over other types due to their consistency, higher drug payload capacity, prolonged half-life in the systemic circulation, and sustained drug release [20,21]. They have been utilized in diverse drug delivery systems such as colonic, nucleic acid, ocular, oral, pulmonary, nasal, mucosal, gene, vaccine, and vaginal delivery, as well as in cancer treatment [14]. One key focus for researchers is minimizing the initial burst release, as a rapid release can lead to drug concentrations that are close to or exceed toxic levels [22]. Innovative methods for encapsulating or binding hydrophobic and hydrophilic compounds, including natural substances, have broadened their applications in biotechnology [23]. The interaction between CS and drugs in these systems involves electrostatic interactions, hydrogen bonding, hydrophobic interactions, and physical adsorption, with non-covalent binding being preferable for small-molecule drugs [24].

In clinical nanomedicine, significant challenges remain, particularly regarding drug loading efficiency [25]. Two crucial parameters in nanomedicines are drug loading content and efficiency [26]. Reevaluating the efficiency of drug loading and encapsulation highlights the need for improvement strategies, given that most NP systems still exhibit relatively low drug loading. This review aims to summarize and compare these different loading strategies for creating NPs with high drug loading. Additionally, this review discusses factors affecting drug loading, providing valuable guidelines for future CSNPs’ development. We examine how advancements in NPs’ design can overcome heterogeneous barriers in drug loading, thereby enhancing efficacy in applications.

## 2. Chitosan Nanoparticles

### 2.1. Progress in Chitosan Nanoparticles

Chitosan (CS) is a biopolymer noted for its biodegradability, bio-compatibility, and lower toxicity [27,28,29,30]. However, a significant challenge to its use is its solubility, which is limited to acidic mediums. The abundant amino and hydroxyl groups in CS are prime targets for chemical modifications aimed at improving nanoparticle solubility. The degree of deacetylation and the molecular weight of CS greatly impact its physical and chemical characteristics. These include its emulsification capacity, aggregation activity, rheological behavior, and overall physicochemical properties. To improve these attributes and extend its range of applications, CS can be modified by adding various functional groups creating functionalized CS derivatives. [31]. The primary goal of these modifications is to improve CS’s solubility, thus expanding its applications [13]. As a natural linear cationic polysaccharide, CS shows great potential for biomolecular delivery [32] to encapsulate a wide range of drugs [14]. Compared to biodegradable polymers like polylactic acid (PLA), poly(lactic-co-glycolic acid) (PLGA), and polycaprolactone (PCL), CS is renewable, unique functional properties, environmentally friendly, and offers superior mucoadhesive properties [17,33]. These attributes make CSNPs ideal for advanced drug delivery systems and other biomedical applications.

**Figure 1:**
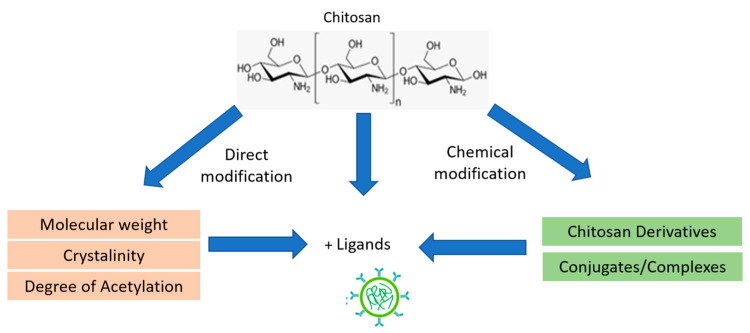
Schematic illustration functionalization of CS [13].

CS, a versatile biopolymer, can undergo a range of modifications to enhance its properties for various applications. These modifications include direct alterations such as reductions in molecular weight, crystallinity, or degree of acetylation. The presence of reactive amine and hydroxyl groups on the CS backbone facilitates numerous chemical modifications, leading to the development of CS derivatives that have gained increasing popularity in research. In the pharmaceutical industry, CS derivatives are commonly synthesized through processes such as acylation, carboxymethylation, quaternization, and thiolation. These modifications confer unique properties to CS, such as improved solubility, enhanced mucoadhesion, and increased bioavailability, which are beneficial for drug delivery systems. Furthermore, CS can be conjugated with other polymers, such as alginate [34], β-cyclodextrins [35], polyphenols [36], gluten [37], catechol [38], etc., to form polyelectrolyte complexes or hydrogels. These conjugates enhance the mechanical strength, stability, and controlled release properties of the resulting material. The modification pattern of chitosan is illustrated in Figure 1 [13,39].

CS’s stability in neutral and basic aqueous solutions can influence its biodegradability, but this characteristic can be advantageous for biomedical applications requiring prolonged stability. The stability supports controlled or sustained drug release, enhancing therapeutic efficacy. Moreover, CSNPs can be engineered to degrade specifically in the acidic environments of certain tissues, such as tumors, ensuring stability during systemic circulation while breaking down where needed. The Warburg effect and resultant acidic environment play a significant role in tumor biology, influencing growth, invasion, and therapeutic responses. Despite its stability in various conditions, CS’s high biocompatibility remains a significant benefit, making it suitable for drug delivery and tissue engineering [40,41,42].

CS can be degraded through various mechanisms—acid hydrolysis, oxidative-reductive processes, nitrous acid depolymerization, ultrasonic degradation, and enzymatic degradation [43]. The degradation half-time and metabolic processes of CSNPs can vary based on several factors, including their size, degree of deacetylation, molecular weight, and the specific environment in which they are used. The degradation half-time of CSNPs can range from days to several weeks. This variability is influenced by factors such as size and surface area, where smaller NPs with a larger surface area may degrade faster than larger ones. Additionally, a higher degree of deacetylation generally leads to slower degradation rates, and higher-molecular-weight CS degrades more slowly than lower-molecular-weight CS. Environmental conditions, such as pH, temperature, and the presence of enzymes, can also significantly affect the degradation rate [44,45]. The patterns of CS degradation with hydrodynamic cavitation were mainly random and central cuts, which indicates that the CS degradation with hydrodynamic cavitation was mainly caused by chemical and mechanical effects [46], structural similarity to natural glycosaminoglycans, and degradation by enzymes such as chitosanase and lysozyme [47].

The primary metabolic process for CS degradation involves enzymatic hydrolysis. CS is mainly degraded by lysozyme, an enzyme present in human tissues and fluids, which hydrolyzes the β(1→4) glycosidic bonds in CS, leading to the formation of chitooligosaccharides and glucosamine [47]. The resulting degradation products, such as glucosamine, are absorbed and can enter various metabolic pathways. Glucosamine is a naturally occurring amino sugar involved in the biosynthesis of glycosylated proteins and lipids [48]. Once absorbed, glucosamine can be utilized in the synthesis of glycosaminoglycans, which are essential components of connective tissues, or it can be further catabolized into smaller molecules that enter the general metabolic pool [49]. Any non-absorbed fragments or metabolites are excreted via the renal system [50].

Research has demonstrated that while CSNPs generally exhibit low toxicity, careful evaluation is necessary to ensure their safety. Comprehensive toxicity assessments—including evaluations of cytotoxicity, hemocompatibility, immunogenicity, and long-term effects—are crucial for confirming that CSNPs are safe and effective for clinical applications [12,51].

### 2.2. Preparation of CSNPs

NPs are dispersed particulates or solid particles ranging in size from 1 to 1000 nm [52,53,54]; some references give a size range of 1–100 nm [55,56]. The characteristics of CSNPs vary significantly based on preparation and surface modification procedures. Various chemical and physical methods have been developed for their formation, including ionic cross-linking, covalent cross-linking, reverse micellar, coacervation/precipitation-based methods, and emulsification [57].

#### 2.2.1. Ionic Cross-Linking Method

The ionic cross-linking method utilizes CS or its derivatives aggregated with oppositely charged macromolecules or with a cross-linking agent like tripolyphosphate (TPP). This process, also known as ionic gelation, forms gels due to ionic linkage. CS-tripolyphosphate ionotropic gelation, a variant of this method, is commonly used for preparing CSNPs. In this approach, CS is dissolved in an acidic solution, and then TPP is gradually added with stirring. This process facilitates the formation of NPs through interactions involving ions [58,59]. Cross-linking CS is necessary for better control over NPs shape and size [20]. The size of CS/TPP NPs initially decreases, stabilizes, and then increases due to swelling and aggregation over time. Swelling is caused by osmotic water influx, and aggregation occurs during storage. Initial nanoparticle sizes and storage conditions influence these variations. Larger NPs decrease in size, while smaller ones increase in a phosphate buffer (pH 7.5 at 25 °C) over 10 days due to protonation or deprotonation of CS molecules. These size changes can be classified into instantaneous, aging, and swelling/aggregation stages over a 97-day storage period [60]. Lazaridou et al. showed that the high solubility of deferoxamine mesylate (DFO) affects the entrapment efficiency negatively. As the drug loading increases with more DFO, the entrapment efficiency decreases because DFO’s high solubility makes it harder to keep the drug encapsulated within the NPs [61].

#### 2.2.2. Covalent Cross-Linking Method

This method involves the creation of covalent bonds between CS or its derivatives and a functional cross-linking agent. Typical agents used include polyethylene glycol, glutaraldehyde, or monofunctional agents [62].

#### 2.2.3. Reverse Micellar Method

This method involves creating a water-in-oil microemulsion by adding an aqueous CS solution to an organic solvent with a surfactant while simultaneously agitating. Extra water is introduced to keep the mixture in an optically transparent micro-emulsion phase, with the quantity of water influencing the size of the NPs. The process includes incorporating a cross-linking agent within the microemulsion. NP formation is initiated by applying an external stimulus like heat or ultrasound to the microemulsion [63,64].

#### 2.2.4. Precipitation/Coacervation

This method leverages the physicochemical properties of CS, particularly its insolubility in alkaline pH, which induces precipitation. A solution of CS is atomized into an alkali solution using a compressed air nozzle, resulting in the formation of coacervate droplets. The resultant particles are then isolated and purified through filtration or centrifugation, followed by washing with hot and cold water. This technique is commonly employed for preparing CS-DNA NPs [65,66]. In another approach, CS and a counterionic polymer, such as alginate, are mixed in an aqueous solution. A pH change or salt addition triggers coacervation, leading to CSNP formation. Encapsulation inside polymer particles is a way to protect active pharmaceutical ingredients (API) against their degradation and/or to control their release [67].

#### 2.2.5. Emulsion Droplet Coalescence Method

This method combines emulsion cross-linking and precipitation principles. Two emulsions are prepared: one containing CS and the drug in liquid paraffin oil and the other containing aqueous CS solution with NaOH in liquid paraffin oil. Upon mixing under high-speed stirring, droplets form, collide, and coalesce, leading to the precipitation of CS droplets and the formation of small-sized particles. In an alternative method, a CS solution is emulsified in an oil phase under high-speed stirring. The resulting emulsion undergoes ultrasonication to break the droplets and form CSNPs [5]. Nanoemulsions have been extensively studied as delivery systems for drug and functional compounds [68], and they offer a practical solution for encapsulating, protecting, and delivering essential oils [69].

## 3. Drug Loading

### 3.1. Drug Loading Capacity in Nanoparticles

The drug loading content indicates the mass ratio of drugs to nanocarriers, whereas drug loading efficiency measures the effective utilization of drugs during the preparation process. Achieving high drug loading content is generally harder than high efficiency. Physical and electrostatic adsorption often lead to low efficiency, whereas crystallization and covalent and coordinate bonds usually yield high efficiency [70,71,72]. High-content nanomedicines are typically prepared using high-efficiency processes [26].

Here, the drug loading and encapsulation efficiency are defined as follows (using Formulas (1) and (2)):

(1) $$\mathrm{Entrapment}\;\mathrm{efficiency}\;\left(\%\right)=\frac{\mathrm{mass}\;\mathrm{of}\;\mathrm{mangostin}\;\mathrm{present}\;\mathrm{in}\;\mathrm{nanoparticle}\;\left(\mathrm{mg}\right)\;}{\mathrm{mass}\;\mathrm{of}\;\mathrm{mangostin}\;\mathrm{used}\;\left(\mathrm{mg}\right)}\times 100\%$$

(2) $$\mathrm{Drug}\;\mathrm{loading}\;\left(\%\right)=\frac{\mathrm{mass}\;\mathrm{of}\;\mathrm{mangostin}\;\mathrm{present}\;\mathrm{in}\;\mathrm{nanoparticle}\;\left(\mathrm{mg}\right)\;}{\mathrm{total}\;\mathrm{mass}\;\mathrm{of}\;\mathrm{nanoparticle}\;\left(\mathrm{mg}\right)}\times 100\%$$

A major drawback of current nanotechnology-based drug delivery systems is the relatively low drug loading, often below 20% for hydrophobic drugs [73]. This requires administering high doses of non-drug materials, which, despite being biocompatible and degradable, can accumulate and become a biological burden over time. High doses of carrier materials can also compromise NPs stability, leading to aggregation and clearance by the mononuclear phagocyte system, reducing bioavailability. Additionally, low drug loading necessitates larger injection volumes and higher NPs concentrations, posing dosage limitations and increasing manufacturing costs [74]. If the carrier material’s space capacity is low, achieving high drug loading content is challenging, even with high drug loading efficiency. For many polymeric NPs, a low rate of drug loading is one of the major pitfalls. Many studies have reported a loading rate of less than 10% [53] due to inert carrier materials requiring high carrier material doses that can cause systemic toxicity. Reducing or avoiding carrier materials can solve this problem and facilitate multidrug co-delivery and combination therapy [75,76,77]. Along with customization options for NP-based delivery systems, this format offers an additional benefit in that it encapsulates cytotoxic drug payloads at high loadings while masking their presence prior to targeted delivery [11].

### 3.2. Drug-Loaded Chitosan Nanoparticles

CSNPs enhance drug stability, bioavailability, and controlled release by overcoming barriers and enhancing permeability. They accommodate both water-soluble and water-insoluble drugs [27,39]. Water-soluble drugs are mixed with a CS solution before particle preparation, while water-insoluble drugs or those precipitating in acidic pH are loaded by soaking preformed NPs in a saturated drug solution. Drugs adhere to nanocarriers through covalent bonding or adsorption mechanisms. Drug loading onto CSNPs occurs during particle formation (incorporation) or post-formation (incubation), involving interactions such as hydrogen bonding, hydrophobic interactions, and electrostatic adsorption. Non-covalent binding is generally preferred for small-molecule drugs compared to covalent bonding [24].

**Figure 2:**
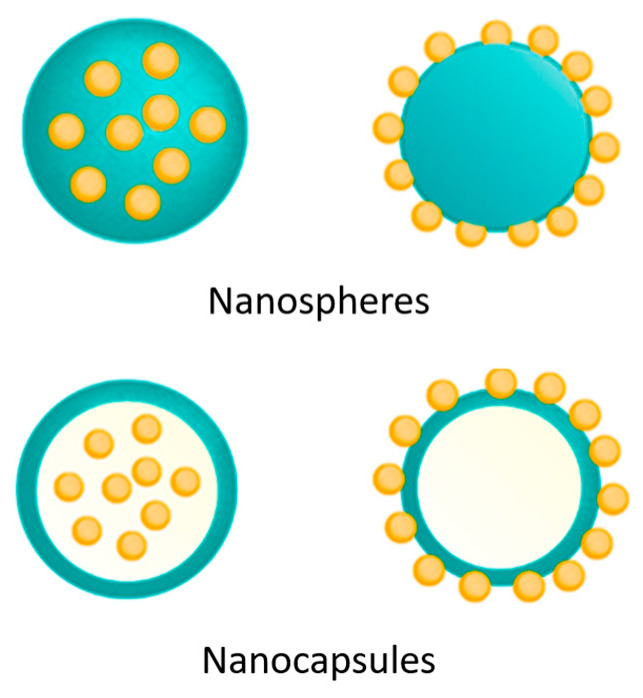
Drug position on CSNPs.

The drug is either immobilized within the matrix or adsorbed onto the surface (Figure 2). Charging efficiency is predominantly influenced by the physicochemical properties of the drug and the method of NP preparation. Optimal loading usually occurs when the drug is included during particle formation, though this can be affected by the preparation method or the presence of additional additives [39].

### 3.3. Factors Influencing Drug Loading in CSNPs

NPs possess the capability to enhance the stability and solubility of encapsulated payloads, facilitate membrane transport, and extend circulation time to enhance safety and efficacy [11]. Polymeric NPs are being engineered in increasingly specified ways so that they can begin to be optimized for drug loading.

#### 3.3.1. Methods of Preparation

The properties of the drug, such as solubility in water, molecular size, and surface charge, directly influence the drug’s ability to be incorporated into NPs matrices [78]. Drugs with lower water solubility tend to exhibit higher drug loading because they are more easily retained within NPs matrices [73,79]. Additionally, small molecular-sized drugs are more readily incorporated compared to larger ones, while the surface charge of drugs can affect electrostatic interactions with NPs, thereby influencing drug loading [39,80]. Hydrophobic therapeutic agents can also be encapsulated within NPs, providing protection from physiological barriers and enhancing their bioavailability [4,8,81].

Meanwhile, NP properties, including the forming materials, particle size, shape, surface charge, and stability, are crucial in determining drug-NP interactions, drug loading, and drug release [82]. For instance, forming materials like CS or polyvinyl alcohol impacts stability and drug interactions within NPs, whereas particle size and shape influence surface area and drug distribution within the delivery system. However, the nanosizing of drug particles has been proven to greatly enhance drug dissolution rate and apparent solubility [83]. The NPs made from double emulsion evaporation also have several drawbacks, such as larger particle size and low drug loading efficiency [53].

#### 3.3.2. Surface Modification

Surface modification of CS is a crucial strategy in the development of NPs for effective and stable drug delivery [13,27,39,64,84,85]. Functionalization of CS surface through chemical modification with functional groups such as thiol, carboxyl, or hydrophilic polymers can significantly enhance CS’s interaction with drugs and the efficiency of drug entrapment within NPs matrices. These functional groups enable CS to interact more strongly with drugs through covalent bonding or hydrogen interactions, thereby strengthening the binding of drugs into NPs [27,84,86].

The use of modified CS, such as quaternized CS or low-molecular-weight CS, is also an effective approach. Quaternized CS exhibits a higher positive charge, enhancing its ability to capture and retain negatively charged drugs [87,88,89]. Meanwhile, low-molecular-weight CS enhances molecular mobility, facilitating the penetration of drugs into NPs matrices more efficiently [89,90].

#### 3.3.3. Synthesis Conditions

Determining the optimal ratio between CS and the drug is highly important. This ratio influences CS’s ability to form a stable and efficient matrix for capturing the drug. By adjusting this ratio appropriately, the capacity for drug loading and the efficiency of entrapment within NPs can be enhanced [45,91,92,93].

Controlling the pH of the medium during NP formation also has a significant impact. Solution pH affects the surface charge of CS, which can influence its interaction with the drug and the stability of the resulting NPs. Selecting the right pH can maximize the desired chemical interactions between CS and the drug, supporting the formation of stable and effective NPs [94,95,96,97].

Optimizing the concentration of polymer and drug in the solution is another critical step [14,27,45]. The correct concentration of each component ensures that NPs form effectively while minimizing raw material loss and maximizing material efficiency.

#### 3.3.4. The Selection of Solvents

The selection of appropriate solvents is critical for developing effective and stable drug delivery NPs. Solvents dissolve both the drug and the NPs matrix, like CS, and their properties significantly impact the final product. A well-chosen solvent should completely dissolve all components without harming their structure or chemical properties [98,99]. Solvent mixtures, particularly those combining organic solvents with water, are effective strategies for enhancing drug solubility and achieving a more uniform distribution within the CS matrix. These mixtures also enable precise control over the size and shape of the resulting NPs [100,101]. However, CS faces challenges due to its limited solubility in both water and most organic solvents, which complicates its practical applications. Traditional solvents like aqueous acetic acid are commonly used to dissolve CS, while an aqueous alkali/urea solution is effective for dissolving natural polysaccharides at low temperatures. Mixed solvent systems of an acetic acid aqueous solution with different imidazolium ionic liquids were developed for successful CS dissolution at room temperature [101,102]. In ionic liquids, both cations and the acetate anion of ionic liquids (Ils) play significant roles in the CS dissolution process by the disruption of the inherent hydrogen bonds of CS. Examples of ILs used are 1-butyl-3-methyl-imidazolium acetate, 1-allyl-3-methylimidazolium acetate, and 1,3-70 dimethylimidazolium acetate [103]. Quaternization of CS conserves its positive charge at neutral pH, thus increasing solubility significantly in a much broader pH and concentration range compared to unmodified CS [51].

#### 3.3.5. Drying Techniques

In the pharmaceutical industry, the quality of the final product cannot be compromised under any circumstances. Stability is the main key feature that determines the product’s quality, safety, and efficacy throughout its shelf life [104]. Drying techniques are a crucial step in nanoparticle development for drug delivery, ensuring optimal physical characteristics and efficiency. Selecting the right method is essential to maintain desired properties and prevent degradation or aggregation [104,105].

Freeze-drying can produce highly stable solid-state products [104]. Freeze-drying has been considered a good technique to improve the long-term stability of colloidal NPs [106]. Freeze-drying, a popular technique, involves freezing the nanoparticle suspension under a vacuum and then sublimating the solvent. This gentle process minimizes aggregation and preserves the porous structure, which is important for efficient drug entrapment within the nanoparticle [106].

Spray drying is another common choice [107]. Here, the suspension is sprayed into hot air to rapidly evaporate the solvent. This technique offers better control over particle size and drug distribution within the nanoparticle matrix, often resulting in more uniform particles [106]. Enzyme encapsulation using spray drying is a versatile strategy to improve enzyme stability in an economical and industrial viable way [106].

#### 3.3.6. Excipients and Stabilizers

Excipients and stabilizers play a critical role in optimizing the stability and efficiency of drug delivery NPs. These additives help maintain the physical integrity of the NPs, prevent particle aggregation, and ensure controlled drug release [108]. Surfactants act as surface tension-reducing agents, aiding in preventing particle agglomeration and maintaining well-dispersed particles [109,110]. The presence of surfactants at the solid interface increased steric stabilization. When the NPs come close to each other, the hydrophobic groups of the surfactant on the surface of the NPs limit the mutual penetration, resulting in steric hindrance and stabilization [110]. Surface adsorbates like ionic surfactants or additives are commonly used to stabilize nanoparticle suspensions, but their effects on the electronic and chemical functionality of nanoparticulate electronic devices remain largely unknown [111].

Cross-linkers form covalent bonds between matrix components, increasing the mechanical strength of the NPs and reducing the possibility of premature drug release [112]. This is important for maintaining NPs stability during storage and transport, as well as preventing [113,114]. Copolymers, on the other hand, can enhance the interaction between the NPs matrix and the drug, promoting stronger and more stable binding [115,116].

The reverse micellar method generates polymeric NPs with a uniform size distribution. Different polymers can be employed to create micelles for drug delivery. Reverse micelles consist of stable liquid mixtures of water, oil, and surfactants governed by thermodynamics [93]. Bimetallic NPs were synthesized via single-step electrodeposition at a constant potential, eliminating the necessity for reducing agents, stabilizers, or surfactants.

#### 3.3.7. Drug Incorporation

In situ drug loading incorporates the drug directly into the NPs matrix during formation. This method offers maximum drug loading efficiency, ensuring a high percentage of the drug is encapsulated. Second, it promotes the even distribution of drug molecules throughout the matrix, preventing aggregation and ensuring consistent release [117,118].

Controlled stirring speed during NP formation is equally important. It influences particle size, size distribution, and how the drug is distributed within the NPs. The optimal stirring speed achieves a uniform particle size distribution, minimizing variations in drug release and promoting even drug distribution throughout the NPs, maximizing delivery effectiveness [119,120].

The three main strategies for producing NPs with high drug loading capacity are as follows:

1. Post-loading

The post-loading strategy entails initially creating core–shell nanocarriers and subsequently loading the drug to achieve highly drug-loaded NPs. Silica gel has been used as a porogen in many preparations of porous materials, such as microporous CS membranes and CS microspheres [121]. The material properties of CS, including its porous structure and tunable surface characteristics, facilitate the incorporation of drugs through various interactions. Non-porous CS materials have also been explored for post-loading, with guest molecules incorporated through noncovalent hydrophobic interactions, electrostatic attractions, and hydrogen bonds. Porosity plays an important role in the case of CSNPs, contributing to their formation, stability, and suitable biomedical applications [122], which regulates the transport of molecules, ions, or particles and contributes to their semi-permeability and selectivity [123].

2. Co-loading

The co-loading approach generally involves attaching a drug to a polymer or macromolecule, followed by self-assembly to create drug-loaded NPs. This method includes loading or encapsulating drugs during the formation of NPs. Various systems have been developed, such as pure drugs, drug–polymer conjugates, drug–silsesquioxane conjugates, metal–organic frameworks (MOFs) with incorporated drugs, solid lipids, proteins, and polymers. Covalent bonding is essential for conjugated systems, while hydrophobic, electrostatic, and π-π interactions are important for polymers and proteins [73].

CSNPs can also be tailored for high drug loadings in the co-loading approach, utilizing mechanisms such as covalent bonding, hydrophobic interactions, electrostatic interactions, or other strategies depending on specific application needs. CS’s versatility in drug delivery systems contributes to its role in achieving effective drug loading and controlled release [39,80].

3. Pre-loading

In the pre-loading approach, drug NPs are initially formed, followed by the creation of a protective shell around the drug core. This strategy involves tuning the thickness of the shell to achieve NPs with high drug loading. The core–shell structure offers several advantages: the shell acts as a diffusion barrier to control drug release, protects the drug core from degradation in external environments, and allows for the engineering of release profiles (such as sustained or stimulus-responsive release). Additionally, the shell can be modified by conjugating targeting molecules, linkers, or other functional groups for diverse biological applications, including drug delivery, biosensing, imaging, and diagnostics [73].

Polymer materials are commonly used for the shell in this strategy due to their excellent biocompatibility, biodegradability, and ease of fabrication. CS’s versatility allows it to be integrated into the pre-loading strategy to achieve NPs with tailored drug release profiles, enhanced stability, and specific biological functionalities, thereby contributing to the development of advanced drug delivery systems [13,124].

## 4. Effect of Drug Loading on Delivery System Characteristics

There are several mechanisms underlying the process of drug loading in CSNPs: (1) Electrostatic interaction: CS possesses positively charged amine groups, while many drugs have negatively charged carboxylate groups. This opposite charge allows for electrostatic interactions between CS and the drug, facilitating drug binding to CSNPs [125,126]. (2) Hydrogen bonding: Hydroxyl groups on CS can form hydrogen bonds with hydroxyl groups on the drug, enhancing the drug’s affinity for CSNPs [127,128]. (3) Physical adsorption: Drugs can be physically adsorbed onto the surface of CSNPs through van der Waals interactions [129]. (4) Encapsulation: Drugs can be encapsulated within CSNPs during the NP formation process. The dominant mechanism of drug loading depends on the properties of both the drug and CS used [129]. The influence of drug loading on the characteristics of the delivery system is evident in Table 1.

### 4.1. Physical Properties

Drug loading significantly impacts the physical properties of CSNPs, including particle size, solubility, dispersibility, and zeta potential. The particle size of CSNPs is influenced by the preparation method and the properties of the loaded drug. Ideally, stable CSNPs typically range between 100 and 200 nm in size, with techniques such as sonication, homogenization, and nanoprecipitation employed to control size. However, high drug loading can induce particle aggregation, leading to larger particles [136]. Increased solubility improves NPs stability by preventing aggregation and precipitation. Modifications of CS, such as surfactant addition or surface coating with hydrophilic polymers like polyethylene glycol, further enhance drug solubility. Moreover, drug loading affects the crystalline structure of CS, impacting its overall solubility properties.

Encapsulation efficiency and the drug-to-polymer ratio significantly influence NP particle size. Particle size is crucial for regulating processes like cellular uptake, intracellular transport, and exocytosis, affecting their transepithelial transport capabilities [137]. NPs possess high surface-to-volume ratios and distinctive physicochemical properties such as biochemical, magnetic, optical, and electrical changes at cellular, atomic, and molecular scales [138]. Accordingly, therapeutic NPs smaller than 100 nm exhibit prolonged circulation in the bloodstream. Numerous studies have demonstrated that NPs sized between 20 and 200 nm exhibit enhanced accumulation in tumors, as they evade recognition by the reticuloendothelial system (RES) and filtration by the kidneys [4]. Larger NPs can accommodate higher drug loading due to their increased volume, but smaller NPs offer better cellular uptake and controlled release due to their higher surface area-to-volume ratio. The optimal size for CSNPs in nanopharmacology is crucial for effective drug delivery and clinical applications. NPs in the 50–200 nm range are generally considered ideal, as they balance cellular uptake, biodistribution, and clearance. Specifically, particles around 100 nm are favored because they can cross the blood–brain barrier (BBB), offer a high surface area for drug delivery, and avoid rapid clearance by the lymphatic system [139]. Research has shown that particles sized around 50 nm exhibit superior tumor tissue accumulation and retention, improving efficacy against primary and metastatic tumors [140]. For targeted therapies, NPs should be initially sized at 50–200 nm to achieve enhanced permeability and retention (EPR) in tumors, then reduced to 10–20 nm for deeper penetration and further shrink to pass through cellular barriers [141]. Additionally, microparticles in the 1–1000 µm range are suited for pulmonary deposition, while NPs in the 1–100 nm range are ideal for systemic delivery and targeted therapies. However, while the traditional upper limit for NPs is 100 nm, a broader definition allows particles up to 1000 nm to be classified as NPs [142].

Ensuring the dispersibility of CSNPs in solution is critical for maintaining their physical stability and preventing sedimentation. This is typically achieved by adding surfactants or copolymers, while pH adjustment helps maintain dispersibility across different solvents. Stabilizers are often necessary, especially with high drug loading, to prevent aggregation and ensure uniform dispersion. The Polydispersity Index (PDI) serves as a crucial parameter indicating the size distribution quality of NP suspensions. A PDI below 0.3 and a single peak in the size distribution curve signify monodisperse-sized dispersion [143].

The stability and surface charge of NPs are commonly assessed through electrophoretic mobility to determine their toxicological profile, as unstable NPs may trigger unintended redox reactions by releasing metal ions into the surrounding media [144]. Achieving an optimal zeta potential is critical for effective nanomedicine, as it directly impacts targeted therapy, stability, and drug release characteristics [145]. The zeta potential of NPs, influenced by drug loading, affects their suspension stability [146]. A higher absolute zeta potential value, whether positive or negative, indicates better stability due to electrostatic repulsion. Modifying the surface charge through the addition of charged molecules or polymers optimizes NPs stability in suspension [145].

Khoshnood et al. demonstrated that in composite polymers, swelling from water absorption causes NPs to expand, increasing the cross-linking of the polymers. The rate of water uptake by NPs is influenced by docosahexaenoic acid (DHA) concentration, medium acidity, and time. At acidic pH levels (pH < 4), protonation of the carboxylic groups in alginate reduces electrostatic repulsion, causing the polymer to shrink. The synergistic effects of amoxicillin and the CS-alginate-docosahexaenoic acid (CA-DHA) formulation increased the biocidal activities against H. pylori infection and improved ulcer healing properties [135]. Chang et al. discovered that the hydrophobicity of cobia liver oil (CBLO), a nutritional supplement that is rich in polyunsaturated fatty acids (PUFAs), such as DHA and EPA, reduces CS swelling and aggregation, resulting in smaller particle sizes. This effect is facilitated by homogenization, which disperses hydrophobic CBLO in solution and forms micelles with surfactants. Composite NPs remained soluble at all pH levels even after a 6 h adjustment period. The presence of hydrophobic CBLO in these micelles prevents CS from aggregating on their surface, thereby forming smaller particles [143].

### 4.2. Drug Release

Drug loading and release are crucial aspects of drug delivery systems, which are influenced by several factors. Drug loading involves incorporating the drug into a polymer matrix or NPs, which affects the release characteristics. Higher drug loading often results in a greater burst effect and faster release, while lower drug loading results in slower release [147]. Understanding the optimal drug loading for a particular application is essential to balance between immediate therapeutic effects and prolonged efficacy. Optimized preparation conditions result in high loading efficiency, the improvement of different pharmaceutical processes, such as bioavailability, and pharmacokinetic profiles, as well as extended drug half-life and decreased frequency of administration [39]. Smaller NPs can extend circulation time, enhancing drug bioavailability and facilitating controlled drug release in vitro [138,148,149]. In contrast, larger NPs, while having longer circulation times, may exhibit reduced accumulation and penetration depths [148].

CSNPs typically exhibit a biphasic release pattern: an initial burst release phase followed by a sustained release phase. The initial burst release can provide a rapid therapeutic effect, while the sustained release ensures prolonged drug availability. The release dynamics in CSNPs are influenced by several mechanisms, including drug dissolution from the NP surface, diffusion through the polymer matrix, and degradation or erosion of the CS matrix over time [45]. Factors such as NP size, surface charge, and porosity also play significant roles in determining the release rate [57]. Research by Lv et al. demonstrated that the in situ crystallization and porous structure of HTCC-NP efficiently encapsulated the drug and improved its release characteristics [130]. PEGylated CSNPs have nanometric size, constricted particle size, proper multiple drug loading, and sustained drug release [150]. CS conjugate polymers synthesized with varying amounts of tetraphenylporphyrin (TPC) show high drug loading and retention capacity due to π–π interactions between the drug and aromatic photosensitizer groups [151]. Large surface area relative to their volume allows for high drug loading and efficient release [27].

The amount of polymer used in nanoparticle formulations also affects drug loading and release characteristics. High polymer concentrations increase nanoparticle density and slow drug release, reducing the burst release effect and providing more sustained drug release. Conversely, low polymer concentrations result in more porous NPs and accelerate drug release, which is beneficial for applications requiring immediate therapeutic effects [45,150].

The degree of cross-linking within the particles significantly affects drug release profiles. Particles with high cross-linking show reduced swelling, limited water penetration, and minimal external drug diffusion [80]. This results in a slower release rate, as increased cross-linking reduces water absorption and restricts drug mobility. Controlling the cross-linking degree allows for the tuning of drug release rates to meet specific therapeutic needs [134].

Various methods can control drug release from NPs, including stimuli-responsive systems that react to environmental changes such as pH or temperature. For example, Sitarek et al. found that the pH significantly impacts the release rate of active substances from NPs, with more noticeable release at pH 7.0 compared to pH 5.8. This pH-sensitive behavior can be particularly useful in targeting specific tissues or pathological sites where pH differs from the normal physiological environment [131].

Overall, controlled drug release from CSNPs is essential to achieve the desired pharmacological effect, improving their solubility and stability and minimizing potential side effects [57,152].

### 4.3. Drug Stability

CSNPs significantly advance drug delivery systems by increasing stability, targeting, and bioavailability [13,39,131,153]. Encapsulating the drug in a CS matrix or NPs protects the drug from enzymatic and chemical degradation, which is important to maintain the drug’s effectiveness over time [153]. The CS matrix provides a physical barrier that protects the drug from environmental factors such as oxygen and moisture, preventing oxidation and hydrolysis, which are common causes of drug degradation [86,154]. Cross-linking CS with substances such as glutaraldehyde or tripolyphosphate can increase the stability of NPs and encapsulated drugs by forming a matrix that is stronger and resistant to degradation [93]. The antioxidant properties of CS provide protection against oxidative damage, help maintain drug integrity, and reduce oxidative stress [28].

The pH-responsive nature of CS can be used to stabilize drugs. CSNPs often maintain stability in a range of pH conditions, which is useful for targeting drugs to specific places in the body that may have different pH levels [155]. The CS-alginate combination results in increased mechanical stability of the gel and resistance to degradation in various biological fluids [156].

CSNPs can provide controlled drug release, thus helping to maintain drug stability over time. By controlling the rate of drug release, this system can minimize the risk of rapid degradation. Dadashi’s research shows that these optimized NPs maintain stability for up to three months, showing the potential for effective pharmaceutical applications. NPs show good biocompatibility, stability, target size 75.60 ± 18.24 nm, and zeta potential 26.70 ± 4.52 mV [59]. Smaller NPs tend to have a larger surface-area-to-volume ratio, which can affect drug stability. Surface modifications with different biomolecules, such as polyethylene glycol, polylactic acid, and polylactic-co-glycolic acid, can significantly increase the physical stability and blood circulation time of CSNPs by evading the immune system and reticuloendothelial system [150].

The size of CSNPs and their ability to protect against physical stability, pH changes, biological fluids, and oxidation are intricately linked to drug loading capacity. Smaller NPs offer a larger surface area for drug interaction, which can enhance drug loading but may compromise stability. Conversely, larger NPs provide better stability and protection against external factors, but this can result in a lower drug loading capacity. The ratio of drug to polymer is crucial in this context: a higher polymer content generally increases protection by forming a more robust matrix that shields the drug from environmental stresses such as pH fluctuations, biological fluids, and oxidative damage. However, this increased protection often comes at the cost of reduced drug loading capacity. Therefore, optimizing the drug-to-polymer ratio is essential to balance the need for effective drug delivery with adequate stability [21,115,152].

### 4.4. Drug Efficacy

In cancer therapy, CSNPs enable precise delivery of anticancer agents directly to tumor cells, thereby maximizing treatment effectiveness while minimizing systemic side effects [116,149]. Similarly, CSNPs are utilized in anti-inflammatory drug delivery systems to deliver therapeutic agents specifically to inflamed tissues, effectively reducing pain and swelling. Moreover, CSNPs serve as effective carriers for vaccines, enhancing immune responses against various diseases by delivering antigens directly to the immune system [157,158].

Incorporating drugs into CSNPs enhances therapeutic activity by protecting them from enzymatic degradation and physical damage, thereby increasing stability and circulation time [153]. CSNPs also improve the solubility and bioavailability of poorly water-soluble drugs [73,159,160,161]. Lv et al. showed that PTX-HTCC NPs are more effective than native PTX, with better cellular uptake and reduced Cremophor EL-associated toxicities compared to Taxol [130].

Furthermore, CSNPs can be customized with specific ligands to target particular cells or tissues, ensuring precise drug delivery and minimizing off-target effects. By facilitating targeted delivery, CSNPs enhance treatment outcomes by delivering higher drug concentrations directly to the intended sites of action [3,7,15,53]. However, comprehensive toxicity assessments, including both in vitro and in vivo studies, are essential to evaluate and mitigate potential adverse effects before clinical application. Strategies such as surface modifications and chemical alterations to minimize immunogenic responses further optimize the safety profile of CSNPs for biomedical use [66,74,162].

Core–shell copper oxide–curcumin and zinc oxide–curcumin nanocomposite materials were developed to enhance stability and antibacterial activity, exhibiting superior antibacterial effects compared to standard antibiotics [134]. CS@CBLO NPs demonstrated less oxidation and fewer hydroperoxides and secondary oxidation products after four weeks of storage compared to CBLO. Comprised of CS and fish oil, CS@CBLO NPs possess excellent oxidative stability, making them suitable for functional food and pharmaceutical applications [143]. Both in situ and post-loading processes significantly impact the formation of magnetic NPs, allowing them to form an outer shell and precipitate on the surface of CS beads [133].

## 5. Perspective

Nanotechnology advancements are revolutionizing disease treatment with nanomaterial-based formulations and drugs. Nano-drugs surpass conventional drugs by offering controlled release, improved absorption, bioavailability, and stability. Factors such as NP size, surface charge, and hydrophobicity enhance targeting, reduce doses, and minimize side effects [52,82].

NPs have evolved into multifunctional micro-nano control tools, acting as “functional molecular devices” precisely controllable in liquid environments [163]. Customizable NP designs enhance precision medicine by addressing patient diversity, disease progression, and individual differences. While tailored NPs improve therapy accessibility and efficacy, they necessitate careful consideration of toxicity, safety, and manufacturing techniques [15].

Advancements in nanotechnology have produced various drug-loaded NPs, particularly those with high drug loading. Highly drug-loaded NPs provide numerous advantages but also encounter issues such as limited loading efficiency and rapid initial release. Post-loading is versatile but can lead to unspecific binding and surface adsorption. Co-loading reduces steps but may produce unstable NPs. Pre-loading, suitable for core–shell NPs, enables controlled release and targeted delivery, mainly for hydrophobic drugs.

NPs with high drug loading show promise, but further research is needed to understand their pharmacokinetics, biodistribution, and therapeutic efficacy. Optimal drug loading requires more investigation to balance dosage and side effects. Although high drug loading aims to reduce dependence on drug carriers, it may impact therapeutic outcomes, necessitating further study. Comparative studies on the efficacy of NPs with high versus low drug loading are essential. Despite clinical advancements, challenges in controlled release, surface modification, and tumor site heterogeneity persist. The recent FDA approval of NPs is encouraging, but additional research is crucial to overcome obstacles and enhance the effectiveness of nano-based therapies.

In improving drug loading, there are other alternative NPs, such as nanogels, which, with their cross-linked hydrophilic polymer networks, offer superior drug loading capacity compared to conventional formulations. The high drug loading efficiency is due to its large surface area and porous structure, thereby enabling effective drug encapsulation via physical, electrostatic, or chemical means [164]. In addition, Ada mesoporous silica NPs have significant advantages due to their large surface area, making them excellent for drug adsorption and loading [53].

NPs are increasingly demonstrating potential in drug delivery systems; however, their clinical application is limited by several drawbacks, including toxicity, high material costs, and laborious preparation procedures. Conventional methods for synthesizing CSNPs often face challenges in controlling particle size, morphology, and scalability. There is a need for a faster, simpler synthesis approach that ensures consistency and reproducibility [59]. CSNP biofabrication addresses these challenges through a one-step process that is environmentally friendly, non-toxic, and energy-efficient [57].

CS is generally considered biocompatible due to its natural origin and low toxicity [165]. However, its use as a polycationic carrier can present challenges, such as cytotoxicity and inflammation, particularly in intravenous applications. Polycation-coated NPs can stimulate macrophages to produce elevated nitric oxide levels and increase the immunogenicity of peptides [166], and polyelectrolyte solutions are more complex due to variable interactions compared to non-charged polymers [167]. Despite these issues, CS demonstrates significant anticancer properties by inhibiting tumor cell growth, angiogenesis, and metastasis, and it can activate innate immune responses by enhancing interactions between dendritic cells and natural killer cells [165,168]. Additionally, CSNPs generally show low cytotoxicity, regardless of particle composition or testing methods [51].

Next-generation NPs are being designed for sophisticated functionality and large-scale production, aiming to enhance disease treatment without compromising efficacy [169]. Developing a new reactor design with high efficiency can produce smaller NPs (~40–60 nm) with low polydispersity and increased productivity [14].

## 6. Conclusions

Drug loading and release are pivotal components of CSNP drug delivery systems. These processes are affected by numerous factors, including the physicochemical properties of the drug, the carrier matrix, the interactions among the drug, matrix, and environment, as well as the characteristics of the NPs, loading methods, and preparation techniques. Understanding the relationships between physical properties, drug release, drug stability, and drug efficacy is essential as they directly impact each other. Further research in these areas will enhance our understanding and ability to tailor drug delivery systems for specific therapeutic applications [77].

## Figures and Tables

**Table 1:** Drug loading in chitosan nanoparticles.

Nanocarrier	Drug	Drug Loading	Physical properties	Drug release	Stability	Efficacy	Ref.
N-((2-hydroxy-3-trimethylammonium) propyl) CS- chloride (HTCC) NPs	Paclitaxel	Co-loading: NPs had a narrow size distribution and high loading efficiency due to the homogeneous distribution of PTX nanocrystals.	d = ~130 nm; narrow size distribution; high loading efficiency owing to the homogeneous distribution of PTX nanocrystals.	The hydrophilic matrix and porous structure of the NPs accelerated degradation and enhanced drug release.	The loading of the drug was directly affected by the stability of the double emulsion, so the choice of emulsifier was crucial.	The PTX-HTCC NPs was more effective than native PTX due to better cellular uptake and caused fewer side effect toxicities than Taxol.	[130]
CS-TPP	*Ginkgo biloba* extract.	Co-loading: The average encapsulation efficiency in CSNPs from five measurements was 53.93 ± 1.62%.	Some NPs are spherical with regular edges, while others are irregular with uneven edges.	pH influences the release rate of the active substance from NPs (water, pH 7.0; saline, pH 5.8).	The results show successful encapsulation of GBE extract in CSNPs. Encapsulating plant extracts with natural polymers enhances stability.	Ginkgo biloba extract showed stronger cytotoxicity on the PEA1 cancer cell line, which was enhanced by encapsulation in CSNPs.	[131]
Porous hydroxyapatite–gelatin (Hap–Gel) composite microsphere coupled with CS.	Doxorubicin	Co-loading: Drug loading increases with higher CS concentrations from 13.95 ± 0.29 to 19.88 ± 0.01. CS’s abundant hydroxyl groups enhance entrapment efficiency from 70% to 99% when combined with Hap-Gel and DOX via hydrogen bonds.	Nanosized HAp crystals and gel polymers form porous microspheres with a surface area of 158.64 m²/g, pore sizes of 3–150 nm, and a pore volume of 0.4915 cm³/g.	CS prolongs the release period while maintaining DOX’s therapeutic effectiveness without affecting it after loading.	Formulations with CS showed 21% drug release after 24 h for DL5. DOX on HAp-gelatin microspheres dissolved easily in PBS, indicating weak bonding. Burst concentration increased as CS decreased.	Cell viability with DOX–Chi/HAp–Gel on day 5 is 57.64%, similar to DOX alone.	[132]
CSNPs	Epidermal growth factor (EGF)	Co-loading: With a high encapsulation efficiency of over 90%, CSNPs exhibited high encapsulation efficiencies across various ratios, correlating closely with CS content.	d = 63.5 to 127 nm; V = +35 to +40 mV. The NPs were spherical, distinct, and regular.	NPs at a 2:1 CS/EGF ratio released 80% of encapsulated protein in 12 h.	Cell proliferation studies showed that NPs preserved EGF functionality at physiological pH.	Significantly accelerated wound closure, re-epithelialization, and collagen deposition	[32]
CS/PEG-loaded Fe_2_O_3_ NP microbeads.	The hydrogels loaded with Fe_2_O_3_ by in situ process.	Post-loading: NPs loaded through an ionotropic process preferentially precipitate on the surface of formed beads.	The CS micro-beads exhibit a smooth surface and a regular, spherical structure. D = 600 ± 50 μm.	-	Surface morphology was influenced by hybrid polymer compositions and variations in the loading process, affecting all roughness parameters.	Nanostructured Fe_2_O_3_ NPs enhance the properties of the polymer blend and improve X-ray radiation shielding.	[133]
Curcumin-β-cyclodextrin inclusion complex	Sodium alginate/CS (CMx-loaded SA/CS) bilayer hydrogels.	Pre-loading: The high content of Ca2+ caused the highly dense polymer chain. The weight of CMx-loaded SA hydrogels cross-linked with 0.05%, 0.10%, and 0.20% w/v.	Two layers showed uniform binding between SA and CS via electrostatic interactions (-RCOO⁻ groups in SA and -NH3⁺ in CS), ensuring close combination.	The cumulative release profiles of CM from CMx-loaded SA hydrogels showed three stages: burst release (0% to ~4% in 30 min), gradual release (~4% at 30 min to ~5% at 12 h), and sustained release (up to ~6% at 48 h).	The CMx-loaded SA/CS bilayer hydrogel improves stability.	The CMx-loaded SA/CS bilayer hydrogels exhibited inhibition against both Gram-negative (Escherichia coli) and Gram CMx-loaded SA/CS bilayer hydrogels were non-toxic to NCTC clone 929 cells and normal human dermal fibroblast cells.	[134]
Docosahexaenoic acid (DHA)-loaded CS/alginate NPs	Amoxicillin-docosahexaenoic acid.	Pre-loading: The spherical shape is attributed to a hydrophobic group (DHA) in the structure. Smaller NPs were obtained using an oil-in-water micelle structure, ensuring homogeneous dispersion of DHA.	Incorporating DHA increased the encapsulation efficiency of AMX to 76%, leading to a reduction in particle size.	AMX and DHA exhibited similar release profiles at pH 4.0 and pH 2.5 but significantly lower release at pH 7.0 (*p* = 0.029 for AMX, *p* < 0.001 for DHA compared to pH 4.0).	Improved drug delivery and stability of encapsulated AMX are indicated.	CA-AMX-DHA showed stronger activity against H. pylori than CA-AMX, CA-DHA, and AMX alone. In vivo, a lower effective dose of AMX was observed with DHA, indicating improved drug delivery and stability.	[135]

## Data Availability

The data that support the findings of this study are available on request from the corresponding author.
